# A randomised controlled feasibility trial of music-assisted language telehealth intervention for minimally verbal autistic children—the MAP study protocol

**DOI:** 10.1186/s40814-021-00918-9

**Published:** 2021-10-01

**Authors:** Tim I. Williams, Tom Loucas, Jacqueline Sin, Mirjana Jeremic, Georgia Aslett, Melissa Knight, Sara Fincham-Majumdar, Fang Liu

**Affiliations:** 1 School of Psychology and Clinical Language Sciences, University of Reading, Reading, UK; 2 School of Health Sciences, City University of London, London, UK; 3 Wellington College, Wokingham, England, UK

**Keywords:** Autism spectrum disorder, Language intervention, Music-assisted programmes (MAP), Treatment-as-usual (TAU), Minimally verbal, Telehealth, Parent-mediated, Social communication

## Abstract

**Background:**

About 30% of children with autism spectrum disorder (ASD) do not develop functional speech and remain non-verbal or minimally verbal even after years of speech, language and educational interventions. A wide range of interventions have been developed for improving communication in ASD, but none have proved effective in eliciting functional language in ASD children. Research has found that people with ASD are more likely to have perfect pitch and prefer music to language. Further, it seems that language delay tends to co-occur with better musical skills. Brain imaging research has found that music alongside words increases the attention that people with ASD pay to spoken words.

**Methods:**

In this protocol, we describe our music-assisted programmes (MAP) that will use music to attract the attention of people with ASD to speech. MAP may open the brain pathways to language and therefore help improve communication skills for people with ASD more than standard communication protocols. In particular, we aim to develop and test whether individualised, easily used MAP would increase spoken language in 24–60-month-old, nonverbal or minimally verbal children with ASD. We will develop a structured training method, delivered through naturalistic, interactive activities (e.g. songs) to teach language to ASD children. We will test this by comparing two groups: one undertaking music-assisted programmes, and the other receiving speech and language therapy in the way that is recommended in NHS clinics. Participants will be allocated to groups randomly. The feasibility of MAP will be assessed through estimations of recruitment and retention rates, the sensitivity and reliability of the outcome measures, the intensity and frequency of the trial, the usability of the MAP app (beta version), and the burden of the assessments for the children and parents.

**Discussion:**

This feasibility randomised controlled trial will establish the acceptability and estimate the power of the MAP intervention to improve early word learning in children with ASD. In the longer term, this research will help us develop an app for parents or carers of children with ASD to design their own songs and implement their own individualised MAP.

**Trial registration:**

ISRCTN, ISRCTN12536062. Registered on 26 June 2019.

## Background

About 30% of children with autism spectrum disorder (ASD) do not develop functional language and remain non-verbal or minimally verbal even after years of speech, language and educational interventions [1, 2]. Evidence from a systematic review indicates that early intervention for preschool children with ASD can improve language outcomes, but with only a small effect size [3]. When just those children with little or no language are considered, the available evidence is limited and does not indicate that spoken language can be improved [4]. Recent research on the special interests of children with ASD has suggested that it might be possible to use music to motivate language learning [5].

Research has indicated that people with ASD have a particular interest and an advantage in musical skills over typically developing (TD) people [6]. The exceptional skills are widespread including perfect pitch [7], identification of musical chords [8] and melodic contour [9], preference for musical over verbal stimuli [10] and intact perception of emotions in music [11]. Some studies have suggested that musical skills are more pronounced in those with language delay [12] or intellectual disability [13]. Behavioural evidence is supported by recent findings from neuroimaging studies suggesting that language and communication impairments in ASD are associated with reduced streamlines in the left arcuate fasciculus along the fronto-temporal pathway [14]. This is consistent with the finding that the activation of the left inferior frontal gyrus and the functional connectivity between the frontal and temporal lobes were significantly reduced in ASD children during speech stimulation [15]. However, these children demonstrated increased activation of the left inferior frontal gyrus and preserved fronto-temporal connectivity during song stimulation and listening [15, 16].

This evidence supports the use of music as a mechanism for intervention and a review of music therapy for individuals with ASD reported that music-based intervention may improve interaction and social communication, including verbal communication [5]. However, the evidence was limited by the small sample sizes of the included studies. More recently, a review of music-based intervention, including for children with ASD, found most studies measured social communication rather than language outcomes [17]. Where language was a target of intervention, evidence was positive but not conclusive. Thus, basic science and evidence from music-based intervention provide a rationale for the aim of this research to use the musical skills and interests of children with ASD to improve learning of single words.

The main objectives of this trial are therefore (1) to determine the feasibility of carrying out a randomised controlled trial of music-assisted programmes delivered remotely due to the COVID-19 pandemic to preschool children with minimal or no spoken language skills, (2) to provide essential parameter data to calculate the sample size and the effect size estimates of a future full-scale trial, (3) to optimise the music-assisted intervention design through a post-intervention interview study with parents, and (4) to pilot and refine an app available on smartphones for supporting and recording homework sessions alongside the intervention.

## Methods/design

### Study design

The study is a randomised controlled trial comparing speech and language treatment as usual with a music-assisted programme for learning language. The online version of the free open-source Python programme, MinimPy [18], will be used by the PI (FL, who will have no prior interactions with the participants) to assign the participants randomly to either the music-assisted (MAP) intervention or treatment as usual (TAU) group using minimisation randomisation. This method prevents selection bias and minimises the imbalance in prognostic factors of participants between treatment groups [19]. Gender, developmental stage (IQ ≥ 70 or 50–69) and echolalia (present or absent, as reported by the parents) will be used to balance participants between groups.

### Participants

Thirty children with ASD will be recruited. This sample size was determined partly based on sample sizes of previous studies that used a similar design, ranging from six [20], twelve [21], 23 [22], to 50 divided across three groups (18, 18, 14 in each group) [23]. In addition, for a small randomised controlled feasibility trial like the current one, the sample size can be established according to the precision required for the critical objectives (e.g., estimation of recruitment rate), rather than through power analysis [24]. With a sample size of 30, we will be able to estimate a drop-out rate of 20% to within a 95% confidence interval of +/− 14% [25]. Finally, it has been recommended that pilot studies should include 12 per group [26] or 30 or more participants [27, 28] in order to estimate the means and standard deviations of the outcome measures, as well as to facilitate a sample size calculation for the full trial. Thus, a sample size of 30 is deemed adequate for the current study.

Participants will be screened for autism spectrum disorder using the Social Responsiveness Scale-2 (SRS-2) [29]. They will be between 24 and 60 months old, with little or no spoken language, defined operationally as fewer than 20 functional words [30]. Adapting Dawson et al. (2010: e18) [31], the following will be the exclusion criteria:

- A neurodevelopmental disorder of known aetiology (e.g., fragile X syndrome);
- Significant hearing or motor impairment;
- Major physical problems such as a chronic serious health condition;
- Seizures at time of entry;
- History of a serious head injury and/or neurologic disease;
- IQ below 50 as measured by the Vineland Adaptive Behavior Scales – Third Edition [32]; and
- Not meeting the cut-off score for ASD on the SRS-2.

The participants will be recruited from special education preschool provision, National Health Service (NHS—state funded), voluntary groups, social media and privately funded clinics in the UK. The parents of potential participants will be approached by letter or email initially and asked to complete an online questionnaire assessing the child’s eligibility and asking for consent to take part in the trial. All data will be held on a secure password-protected database accessible only to the research team.

### Ethics

Ethical approval was granted by the European Research Council (ERC) ethics review panel (Reference no. MAP, 838787), the NHS (National Health Service) HRA (Health Research Authority) and HCRW (Health and Care Research Wales) Approval service (Reference no. 262697) and the University Research Ethics Committee (UREC) of the University of Reading (Reference no. UREC 19/07). Any changes to the protocol will be submitted to the ERC, NHS HRA and HRCW, as well as the University of Reading Research Ethics Committee.

Since 24–60-month-old nonverbal and minimally verbal children are unable to give informed assent, consent will be sought from their parents, legal guardians, or caregivers. They will be sent via email a copy of the information sheet to read, and then be verbally briefed via video-conferencing through Microsoft Teams by the research speech and language therapist. If they agree for their child/dependent to participate, they will be given a consent form to sign. Children’s assent will be monitored by their behaviour and responses to their parent/carer during the sessions. Sessions will cease immediately if children become distressed.

### Interventions

To ensure social distancing as required by UK COVID-19 regulations, assessments and interventions will be carried out by the parents under online guidance from the research speech and language therapist using Microsoft Teams. The interventions will consist of 36 training sessions of 45 min duration delivered at the rate of two sessions a week for 18 weeks, during which the parent will be coached by the therapist carrying out a range of activities with the child. In addition, parents will be encouraged to carry out a minimum of five 10-min practice sessions with their children per week. Each week, one such session will be recorded and jointly reviewed by the therapist and the parent to enable better tailoring of the treatment.

The MAP intervention will be as follows (Protocol version MAP-005): (1) Naturalistic strategies will be used, such as incidental learning, high-density repetition, time-delay and mand-modelling. (2) For each of the 36 target words, we will create a set of songs providing the contexts where it occurs. During each session, the songs will be played using a computer or a phone, and sung with a range of home-made music instruments such as shakers. (3) The children will be taught to sing the songs, in which the target words will be occurring repetitively, together with other engaging and interactive activities such as dancing, vocalising, improvising, and playing musical games. (4) Parents will be taught strategies including intensive interaction and communication temptations to help them engage with their children. Parents will also be provided with an app (software programme for Android phones) written to provide the target words songs. The app enables tracking of the amount of practice sessions (i.e., without the therapist present) undertaken by each parent-child dyad.

For the treatment as usual (TAU) group, the speech and language therapy will be modelled on that provided in normal clinical practice for young children with little or no spoken language in the University of Reading Speech and Language Therapy clinic but delivered via online methods (Protocol version MAP-005). It will be based on Social Communication Intervention for Preschoolers (SCIP) [33] modified to teach the 36 target words identified. SCIP is a naturalistic developmental intervention for the core features of autism and uses a parent-mediated approach as recommended by the National Institute for Health and Care Excellence [34] guideline on psychosocial interventions for children with autism. The approach teaches parents child-centred, play-based strategies to improve their children’s joint attention, engagement, reciprocal social interaction, communication and language. Video feedback and coaching is used to improve a parent’s synchrony with their child’s interaction, to teach them how to develop reciprocal play routines, and how to provide general language stimulation.

### Feasibility measures

The study is designed to estimate recruitment and retention rates from multiple referral sources. One aspect of these estimations is the feasibility of recruiting a narrowly defined group of preschool children with ASD. The initial screening is carried out by a therapist working jointly with parents to determine the history and skill levels of the child. We will gather information about the proportion of referrals that meet the criteria for inclusion and the effect of intake assessment on retention. Once the caregivers have completed the screening stage, they will then be randomised to the two interventions. Since the MAP intervention is novel, the trial will document changes to the methods and assess the acceptability of the intervention to the caregivers and children. In part, this will be through careful documenting of reasons for withdrawal.

One outcome measure (words learnt from a restricted list, Table 1) may need to be revised in light of evidence about the numbers of words already known by the participants and which words are observed to attract the most attention and learnt most rapidly during the intervention. If words on the target list are already known by the participant, we will provide alternatives from the list in Table 2.

**Table 1:** The 36 target words to be learned during the trial

Mummy	Daddy	Hands	Home	Toys	Book
TV	Park	Ball	Ouch	Cat	Bye-bye
Bed	Food	Red	Green	Happy	Sad
Singing	Stop	Go	Kiss	Play	Sleeping
Hello	Help	Drink	Look	Tired	Hungry
Yes	No	More	Please	Yummy	Thank you

**Table 2:** The list of 35 backup words for children who already know some of the pre-determined 36 target words

Bump	Rainbow	Sky	Bubbles	Belly	Music
Car	Dog	Blue	Yellow	Finished	Rub
In	Outside	Bath-time	Night-time	Clap	Driving
Sitting	Smiling	Yay	Brum	Dancing	Hug
Fall	Grandma	Sister	Brother	Grandpa	Cold
Thirsty	Hot	Feet	Teddy	Water	

Finally, we plan to assess the burden of the follow-up measures through a qualitative study of a subsample (*n* = 6) of the participants. The qualitative study will use semi-structured interviews with a topic guide of ten questions/prompts to examine the experiences of participants and their caregivers about the duration, intensity and practicalities of treatment and assessment via video link with the person’s home. Using MS Teams, the interviews will be conducted and video-recorded by TL, with help from a research assistant.

### Baseline and outcome measures

**Fig. 1:**
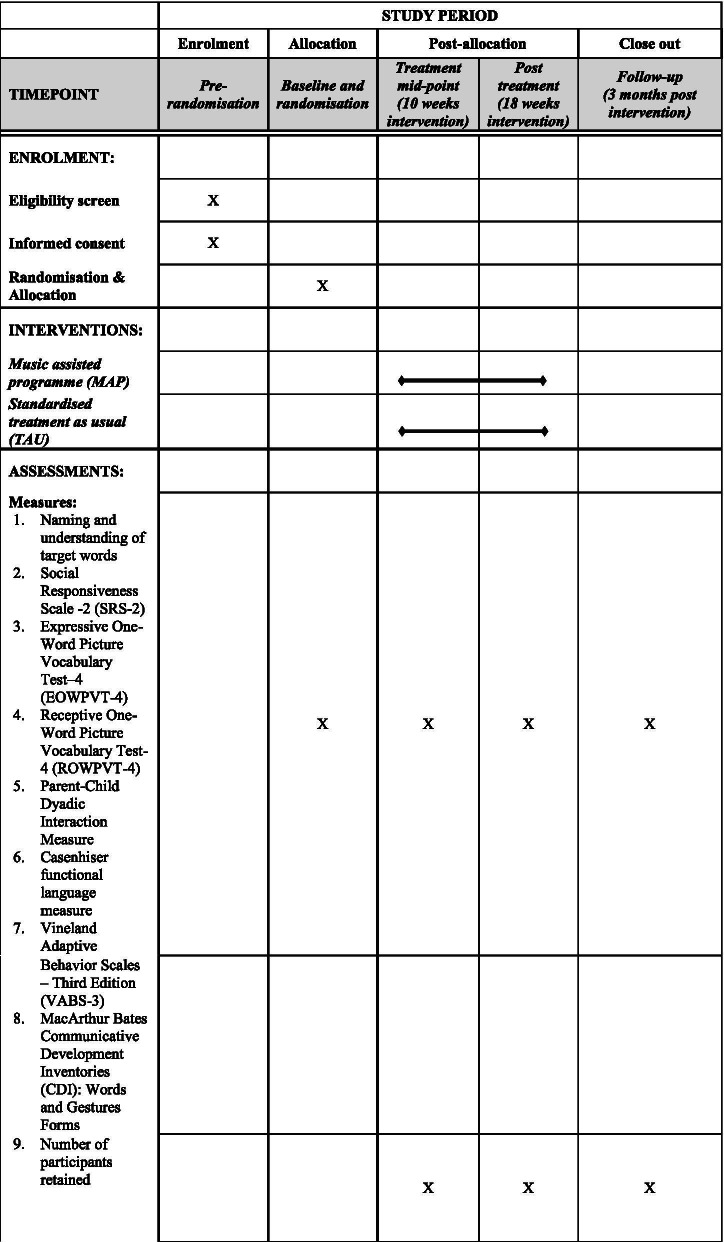
Schedule of data collection

Outcome measures will be collected at four time points: pre-, mid-, post-intervention and 3-month follow-up (see Fig. 1 and the SPIRIT checklist [35] in Supplementary Material). The measures will be administered by the research therapist using telecommunication methods including video conferencing technology to direct the caregiver to manipulate materials appropriately. The measures can be successfully administered with non-verbal and minimally verbal children and can ultimately be easily integrated in clinical and research settings. They will be scored by the research therapist and checked by a research assistant who will be blind to allocation. However, the parent administering the measures will also have been using the intervention methods with their children and thus there is very limited blinding possible in this trial. Demographic details and a medical history will be obtained at intake to the trial. Given the small size of this randomised controlled feasibility trial, there is no need to define primary versus secondary outcomes [24]. Thus, the following outcome measures are listed with no particular order.

1. One key outcome is the expressive (picture naming) and receptive (pointing to correct picture) tasks to assess the production and comprehension of the 36 target words at baseline, post-intervention, and 3-month follow-up. A preliminary list of 36 target words has been drawn up (Table 1), but the protocol includes 35 backup words (Table 2) and allows substitutions of up to 10 words if required by the prior skills of the child;
2. Social Responsiveness Scale-2 (SRS-2) - Participants’ ASD symptomatology will be assessed using the Social Responsiveness Scale-2 [29]. The SRS-2 is a questionnaire that uses a scale of 1–4 (1 = not true, 4 = almost always true) to rate items, with higher scores indicating greater impairment [36, 37]. A designated raw score cutoff value of 70 is considered to have a sensitivity of 0.78 and specificity of 0.94 for ASD [38]. Raw total scores are converted to gender-normed T scores, with a T score of 75 indicating severe impairment. Item-level and full scale (both raw and T scores) scores were included in the analyses.
3. Receptive and expressive language skills will be measured using the Expressive One-Word Picture Vocabulary Test–4 (EOWPVT-4) [39] and the Receptive One-Word Picture Vocabulary Test-4 (ROWPVT-4) [40], and by MacArthur Bates Communicative Development Inventories (CDI): Words and Gestures Forms [41]. The EOWPVT-4 and ROWPVT-4 tests give raw score which can be used to determine standard score, age-equivalent score and percentile rank. The tests are appropriate for use with typical and atypical populations, including those with learning difficulties. The CDI has high parent-teacher agreement for children with ASD (intraclass correlation 95% CI estimates 0.77 to 0.93) [42].
4. The child’s social communication will be evaluated during a 10-min video recorded free play session between the child and their parent. Social communication will be measured using the Parent-Child Dyadic Interaction Measure which has good intrarater (*κ* = .81) and good interrater reliability (*κ* = .80) [43].
5. Functional language will also be evaluated during the 10-min video recorded free play session between the child and their parent. Casenhiser et al.’s (2015) [44] approach to coding language functions will be used which shows good internal consistency (Cronbach’s *α* range 0.830–0.836) as is the validity compared with other measures of communication level. A week-long language diary to be filled out by parents at pre-, mid-, post-intervention and 3-month follow-up will also help determine the child’s progress on language development.
6. Communication, daily living skills, socialisation and maladaptive behaviour will be measured using the comprehensive parent/carer form of Vineland Adaptive Behavior Scales – Third Edition (VABS-3) [32], to be filled out by parents/caregivers. The VABS-3 has high internal consistency (Cronbach’s *α* range 0.86–0.97), good test-retest reliability (corrected *r* values range 0.62–0.92), and good inter-rater reliability (range 0.61 to 0.87). Data show that it has criterion validity for identifying those with intellectual disability.

### Data handling and analysis plan

All electronic data will be stored in a secure password-protected folder to which only the trial team will have access. Video recordings and questionnaire-based outcome measures will be coded and scored by research assistants who are blinded to the study purpose, treatment allocation, and testing order.

We will record and report the participant flow according to CONSORT guideline and produce a CONSORT flowchart [45, 46]. As a feasibility trial, we will report recruitment and retention figures for both MAP and TAU together with reasons for loss of participants. The trial management will consider any adverse effects and use that information to continue or halt the trial. In addition, we will discontinue the trial at the request of the caregivers, ongoing distress on the part of either the child or the caregiver, and if the caregiver takes up an additional language intervention during the trial. We expect to analyse recruitment and retention data using descriptive statistics involving both intention to treat and actual completed participant data. Recruitment and retention will be encouraged by payment of £202.50 ($283.00) to participants families for completing the research. The amount of the missing data will be recorded in order to assess the feasibility of using each measure. An important part of the feasibility is the extent to which it is possible to conduct two training sessions per week and still maximise the homework practice. We will therefore review the uptake/usage pattern of all MAP parent-child dyads to establish the feasibility of delivering 36 sessions over 18 weeks. For the outcome measures, descriptive data will include means and standard deviations for continuous normal data, and medians and inter-quartile ranges for continuous non-normal or discrete data. Standard errors/confidence intervals will also be calculated for continuous normal data or percentages. Statistical analyses will be performed in R [47]. The results from these analyses will provide data for effect size estimation. Together with the trial parameter data (i.e., recruitment, retention, follow-up and completion rate), these data will be used to determine the size of sample necessary to carry out a fully powered randomised controlled trial comparing MAP with SCIP. A preliminary categorical analysis of words learnt may be used to inform the selection of future vocabulary items for a subsequent trial. The numerical data will be analysed blind to allocation. The blinding will be carried out once the data are collected by generating a new series of participant codes, removing data that could lead to identification (TW) and then be analysed by the PI (FL). Anonymised behavioural data and statistical analysis codes will be made available following open access guidelines. The qualitative interview data will be transcribed and analysed using the thematic analysis framework [48] and Nvivo 12 [49].

If during the course of the trial, we make observations which raise concerns about the children’s wellbeing and safety, we will follow the procedures and policies as outlined by the Keeping Children Safe Standards in the EU [50], the Safeguarding Vulnerable Groups Act 2006 in the UK [51], and the University of Reading DBS policy [52] and safeguarding policy [53] to respond to any safeguarding concerns. There will be no data monitoring committee because the number of participants is small and the primary data offer few opportunities for analysis methods. The trial therapists (MJ and MK) are supervised by TL and SF-M as part of the clinic quality control procedures.

### Dissemination policies

The aim of dissemination will be to inform clinicians and parents of the potential usefulness or otherwise of the music-assisted programmes and the app that we are able to develop. This will be achieved through presentations to parent and voluntary groups. Other outputs will include conference presentations for organisations involved in the care and education of preschool children with ASD, and scientific conferences about interventions for ASD. A paper will be written for a peer-reviewed publication which we will make available cost free online.

## Discussion

The current study is designed to examine the feasibility of running a trial to compare music-assisted language intervention with treatment as usual carried out by experienced therapists using telecommunication methods. The aim is to provide data that would allow the design of a fully powered randomised controlled trial with appropriate blinding of outcome assessors.

The prognosis for young children with ASD is significantly affected by their language development [54]. Unfortunately, the literature to date does not clearly recommend the adoption of any one intervention over others for improving language in preschool children [55]. If this trial proves to be acceptable and manageable by caregivers in the community, it will open the way for a fully blinded randomised controlled trial of music as an intervention tool for improving the language skills of children with ASD.

## Data Availability

Anonymised behavioural data and statistical analysis codes will be made available following open access guidelines.

## Abbreviations

ASD: Autism spectrum disorder
TD: Typically developing
MAP: Music-assisted programmes
TAU: Treatment as usual
SCIP: Social Communication Intervention for Preschoolers
SRS-2: Social Responsiveness Scale-2
NHS: National Health Service
ERC: European Research Council
HRA: Health Research Authority
HCRW: Health and Care Research Wales
UREC: University Research Ethics Committee
EOWPVT-4: Expressive One-Word Picture Vocabulary Test-4
ROWPVT-4: Receptive One-Word Picture Vocabulary Test-4
CDI: MacArthur Bates Communicative Development Inventories
VABS-3: Vineland Adaptive Behavior Scales – Third Edition
CONSORT: Consolidated Standards of Reporting Trials
SPIRIT: Standard Protocol Items: Recommendations for Interventional Trials
